# Suicide, Socio-economic Inequalities, Gender, and Psychiatric Disorders Commentary: Educational Levels and Risk of Suicide in Japan: The Japan Public Health Center Study (JPHC) Cohort I

**DOI:** 10.2188/jea.JE20160082

**Published:** 2016-06-05

**Authors:** Eiji Yoshioka

**Affiliations:** Department of Health Science, Asahikawa Medical University, Asahikawa, Hokkaido, Japan

**Keywords:** suicide, socio-economic inequalities, gender, psychiatric disorders

This issue of the *Journal of Epidemiology* includes a report titled “Educational Levels and Risk of Suicide in Japan: The Japan Public Health Center Study (JPHC) Cohort I” by Kimura and colleagues.^1^ These authors investigated the association between education level and suicide risk in a population-based cohort in Japan. They considered educational level to serve as a surrogate marker for socioeconomic status. In total, 21 829 males and 24 327 females were included in the analysis reported in this article, and 218 males and 81 females died by suicide during a median follow-up period of 21.6 years. Their results revealed that, compared with junior high school graduates as a reference category, the hazard ratios in men were 0.91 (95% confidence interval [CI], 0.67–1.22) for high school graduates, 1.01 (95% CI, 0.54–1.90) for junior/career college graduates, and 0.47 (95% CI, 0.24–0.94) for university graduates or those with higher education. For women, the hazard ratios were 0.44 (95% CI, 0.24–0.79) for high school graduates, 0.56 (95% CI, 0.22–1.43) for junior/career college graduates, and 0.55 (95% CI, 0.06–4.03) for university graduates or those with higher education. The authors concluded that high educational levels among Japanese men and women were associated with a reduced risk of suicide, suggesting that higher educational levels have a protective role against suicide risk in the Japanese society.

This is the first large-scale population-based prospective study in Japan to examine the association between educational levels and the risk of suicide after controlling for individual lifestyle and socioeconomic factors. The study is organized well and is important for future suicide-prevention implications in Japan. The results for males in the study are generally consistent with those in previous studies from other countries or regions. However, I think that we need to interpret the results for the female participants more carefully because only a small number of female participants, particularly those with a higher level of education, died by suicide during the follow-up period.

The European Union (EU) Working Group on Socio-Economic Inequalities in Health previously reported a European overview of socioeconomic inequalities in mortality arising from suicide among men and women.^2^ The EU working group used a prospective follow-up of censuses matched with vital statistics in 10 European populations in the early 1990s. The mean follow-up period was 4 years. In this earlier article, level of education and housing tenure were used as socioeconomic indicators. The main findings of this EU study were that socioeconomic inequalities in suicide were pervasive in almost all male populations and that inequalities were far less pronounced (or even reversed, in some cases) in women, particularly when educational status was considered.^2^ The authors of the EU study suggested that this gender difference could be explained by gender differences in health-related and life-threatening behaviors, such as alcohol or drug misuse, which were known risk factors for suicide and were more prevalent among men and among people in lower socioeconomic groups. In addition, it was reported that suicide risks in women resembled those in men more closely where house ownership was concerned rather than education. This may be because house ownership was mostly an attribute of the household and was thus shared by both spouses, whereas education is an individual attribute and thus may be more sensitive to differences in gender.

As is the case with educational level, it has been reported that school performance also affects suicidal behavior differently between males and females. For example, Gunnell et al examined the association of school performance scores at the ages of 16 years and 18/19 years with suicide in a record linkage study of Swedish education, population, and census data with mortality and inpatient registries.^3^ At approximately 16 years, all Swedish children have school assessments in the final year of their compulsory education. At 18/19 years, most Swedish children leave upper secondary school. Data were available for 95 497 males and 91 311 females born in 1972 and 1977, and cases were monitored until December 31, 2005. During the follow-up period, 230 males and 90 females died by suicide. In 16-year-old males, there was a four-fold risk increase among those with the lowest 20% of school grades compared to those with the highest 20%. Similar associations were seen with upper-secondary school performance (age 18/19 years). No association was detected between school performance and suicide risk in females; however, in a sub-group of females (47%) who studied theoretical (more academic) courses in secondary school, there was weak evidence that those with better school performance had an increased risk of suicide. It is possible that these differing associations reflect differences in life chances and satisfaction among men and women in the light of being able or unable to perform well at school. The strong, graded association of education level or school performance with suicide in males and the absence of such an association in females require further investigation to understand preventive implications.

Suicide prevention in psychiatric patients is particularly important because psychiatric disorders, such as clinical depression, alcohol abuse, and schizophrenia, represent the best-known risk factors for suicide and over 90% of individuals who die by suicide have a diagnosable psychiatric illness.^4^ Although the risk of suicide in the general population appears to be associated with low income, unemployment, educational underachievement, and being single, data indicate that the opposite is true among psychiatric patients. For example, Agerbo examined the associations among suicide risk, socioeconomic position, and marital status in psychiatric patients using data obtained by linking Danish population-based registries.^5^ These data included all first-time psychiatric patients aged 16–65 years admitted between 1981 and 1998. They also included administrative longitudinal data on income, labor market affiliation, educational attainment, and marital and cohabitational statuses (96 369 patients, 256 619 admissions, and 2727 suicides). The suicide hazard ratio for patients in the lowest income quartile group versus the highest income group was 0.38, the hazard ratio for the unemployed versus the fully employed was 0.85, the hazard ratio for primary school education group versus postgraduate education was 0.54, and the hazard ratio for divorced patients versus married patients was 0.74. However, psychiatric patients who experienced loss of income, employment, or marriage had a higher risk of suicide.

The Swedish record linkage study^3^ that examined the associations between school performance and suicide also revealed that school performance was not associated with suicide risk in men who developed severe psychiatric illness but that, for women who developed severe psychiatric illness, suicide risk was higher in those who performed better in school at 16 years of age. One possible explanation for these differences in patients with and without psychiatric illness is that the stigma associated with having a severe mental illness is greater in higher earners and more educated individuals. This stigma may increase an individual’s sense of hopelessness and thus increase the risk of suicide. Alternatively, the gap between an individual’s aspirations, based on their education, and their limitations, based on the occurrence of severe mental illness, may be greater in individuals with a higher socioeconomic status. This discordance may increase their risk of suicide. Although redressing the stigmatization of mental illness is a major target for the prevention of suicide, it is not yet evident whether patients who have managed well earlier in life are particularly prone to facing such stigma. Therefore, further research studies are needed to investigate this possibility.

Although the reduction of socioeconomic inequalities represents an important target for promoting suicide prevention efforts throughout the world, predictive models and risk-screening for suicide must account for the context within which risk is assessed. This is because risk factors for suicide can differ between men and women and in people with and without psychiatric illness. Most suicide prevention research in the field of social epidemiology has been carried out in western countries. In April 2016 in Japan, the Revised Basic Act on Suicide Prevention, which promotes suicide prevention research, was implemented. Accumulating scientific knowledge concerning the association between socioeconomic inequalities and suicide risk in Japan will greatly contribute to the development of measures to prevent suicide in Japan as well as the other countries.
